# Teriflunomide shifts the astrocytic bioenergetic profile from oxidative metabolism to glycolysis and attenuates TNFα-induced inflammatory responses

**DOI:** 10.1038/s41598-022-07024-7

**Published:** 2022-02-23

**Authors:** Parijat Kabiraj, Ethan M. Grund, Benjamin D. S. Clarkson, Renee K. Johnson, Reghann G. LaFrance-Corey, Claudia F. Lucchinetti, Charles L. Howe

**Affiliations:** 1 Department of Neurology, Mayo Clinic, Rochester, MN 55905 USA; 2 Translational Neuroimmunology Lab, Mayo Clinic, Guggenheim 1542C, 200 First Street SW, Rochester, MN 55905 USA; 3 Mayo Graduate School Neuroscience PhD Program and Medical Scientist Training Program, Mayo Clinic Graduate School of Biomedical Sciences, Rochester, MN 55905 USA; 4 Division of Experimental Neurology, Mayo Clinic, Rochester, MN 55905 USA; 5 Center for Multiple Sclerosis and Autoimmune Neurology, Mayo Clinic, Rochester, MN 55905 USA

**Keywords:** Glial biology, Molecular neuroscience, Neuroimmunology

## Abstract

Astrocytes utilize both glycolytic and mitochondrial pathways to power cellular processes that are vital to maintaining normal CNS functions. These cells also mount inflammatory and acute phase reactive programs in response to diverse stimuli. While the metabolic functions of astrocytes under homeostatic conditions are well-studied, the role of cellular bioenergetics in astrocyte reactivity is poorly understood. Teriflunomide exerts immunomodulatory effects in diseases such as multiple sclerosis by metabolically reprogramming lymphocytes and myeloid cells. We hypothesized that teriflunomide would constrain astrocytic inflammatory responses. Purified murine astrocytes were grown under serum-free conditions to prevent acquisition of a spontaneous reactive state. Stimulation with TNFα activated NFκB and increased secretion of Lcn2. TNFα stimulation increased basal respiration, maximal respiration, and ATP production in astrocytes, as assessed by oxygen consumption rate. TNFα also increased glycolytic reserve and glycolytic capacity of astrocytes but did not change the basal glycolytic rate, as assessed by measuring the extracellular acidification rate. TNFα specifically increased mitochondrial ATP production and secretion of Lcn2 required ATP generated by oxidative phosphorylation. Inhibition of dihydroorotate dehydrogenase via teriflunomide transiently increased both oxidative phosphorylation and glycolysis in quiescent astrocytes, but only the increased glycolytic ATP production was sustained over time, resulting in a bias away from mitochondrial ATP production even at doses down to 1 μM. Preconditioning with teriflunomide prevented the TNFα-induced skew toward oxidative phosphorylation, reduced mitochondrial ATP production, and reduced astrocytic inflammatory responses, suggesting that this drug may limit neuroinflammation by acting as a metabolomodulator.

## Introduction

Astrocytes are the most abundant cell type in the CNS and are structurally, functionally, and metabolically coupled with neurons. During homeostatic conditions, astrocytes metabolize the majority of brain glucose and exhibit high glycolytic flux to provide bioenergetic substrates to neurons^1,2^. Simultaneously, astrocytes utilize oxidative phosphorylation to metabolize extracellular glutamate, thereby modulating synaptic function, preventing excitotoxicity, and sustaining excitatory neurotransmission through the glutamate-glutamine cycle^1,3–8^. This cycle does not operate stoichiometrically and is used to facilitate diverse metabolic demands within astrocytes. While glucose and glutamate metabolism have been intensively studied in astrocytes, the role of metabolism in other important astrocytic functions, such as neuroinflammatory modulation, are not well characterized.

Astrocytes respond to diverse pathogenic stimuli with a reactive program that involves cellular hypertrophy and profound alteration of the secretome^9^. In addition to producing and releasing inflammatory cytokines and chemokines, astrocytes also engage the acute phase response and release molecules such as lipocalin-2 (Lcn2). Lcn2 is a soluble iron-chelating protein that induces autocrine and paracrine changes in cellular migration and reactivity and drives cell death in neurons and oligodendrocytes^10^. While metabolic changes are a component of the acute phase response^11^, the role of metabolism in the production and release of effectors such as Lcn2 is unknown.

Teriflunomide (TF) is an inhibitor of dihydroorotate dehydrogenase (DHODH)^12^, a mitochondrial enzyme that mediates nucleotide ring formation in the de novo biosynthesis of pyrimidines^13^. TF is used for the treatment of multiple sclerosis, psoriatic arthritis, and rheumatoid arthritis^14^ based on the immunomodulatory effect of inhibiting pyrimidine synthesis in rapidly dividing cells such as activated T cells^15^. While the specific mechanism of action remains unclear^16,17^, the role of DHODH in mitochondrial bioenergetics suggests that TF may function as a metabolic reprograming agent^18^. On this basis, we sought to determine if TF could alter the bioenergetic profile of astrocytes and change the cellular response to inflammatory activation. We hypothesized that TF treatment would constrain the secretion of Lcn2 in response to cellular activation by TNFα via a mechanism involving metabolomodulation.

## Results

### TNFα drives inflammatory signaling and chemokine release in purified primary astrocytes

**Figure 1:**
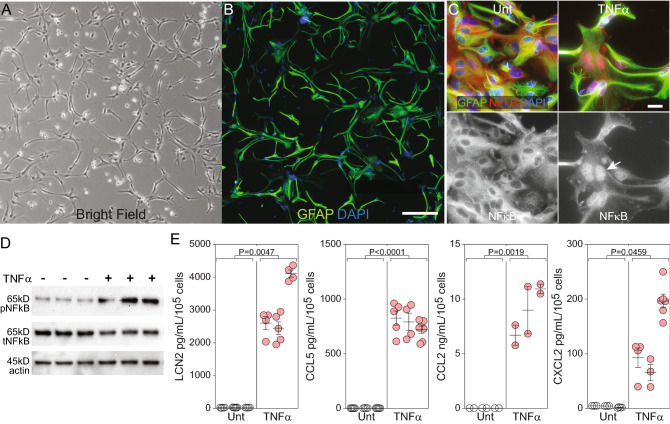
TNFα drives inflammatory reactivity in astrocytes grown under serum-free conditions. (**A**) Bright-field image of purified astrocytes cultured in serum-free conditions for at least 14 days indicating quiescent phenotype. Cells were seeded at 500,000 cells/well in 6-well plates. Scale bar = 200 µm. (**B**) Fluorescent image of astrocytes (GFAP = green; DAPI = blue) verifying resting morphology. Scale bar = 200 µm. (**C**) Fluorescent images (GFAP = green; NFκB = red; DAPI = blue) of resting astrocytes under untreated conditions (Unt) or following stimulation with TNFα (10 ng/mL for 1 h) indicate nuclear translocation (white arrow) of NFκB consistent with signaling via this pathway. Scale bars = 20 µm. (**D**) Western blot analysis of cell lysates indicating that TNFα (10 ng/mL for 1 h) stimulation of the quiescent astrocytes induced robust phosphorylation of NFκB. Actin is shown as a loading control. Immunoblots derive from the same membrane. Uncropped blots are available in the Supplementary Information. (**E**) Resting astrocytes were left unstimulated (Unt) or treated with TNFα (10 ng/mL for 24 h). Supernatants were analyzed by ELISA to measure secretion of Lcn2, CCL5, CCL2, and CXCL2. Data are normalized to pg/mL per 10^5^ input cells. Each symbol represents one well from 3 separate experiments, shown in nested layout. Mean ± SEM is shown for each biological replicate. P-values were calculated using mixed model nested t-tests. Western blots and fluorescent images are representative of at least 3 separate experimental replicates.

In order to obtain quiescent astrocytes without microglial contamination, mixed glial cultures were treated with clodrosome^19^ and then grown under serum-free conditions for at least 14 days. Culture purity was assessed by flow cytometry (98.8% ± 0.2% ASCA-2^+^ astrocytes; 0.3% ± 0.1% O4^+^ oligodendrocytes; 0.06% ± 0.04% CD11b^+^ microglia; 0.9% ± 0.3% unlabeled other). Removing serum from the culture system was critical to preventing a spontaneous reactive state^20^ and for establishing reproducible baseline bioenergetic profiles. Under resting conditions, the astrocytes exhibited an elongated morphology (Fig. 1A) marked by a low-complexity GFAP^+^ ramified phenotype (Fig. 1B). Stimulation of these quiescent astrocytes with TNFα (10 ng/mL) for 1 h induced NFκB nuclear translocation (Fig. 1C) and phosphorylation (Fig. 1D), indicating inflammatory pathway activation. TNFα-induced NFκB phosphorylation was four times higher relative to untreated conditions (*P* = 0.0046). TNFα stimulation for 24 h also induced secretion of Lcn2 (116-fold), CCL5 (226-fold), CCL2 (642-fold), and CXCL2 (36-fold) (Fig. 1E). We conclude that TNFα induces an inflammatory phenotype in pure cultures of astrocytes grown under serum-free conditions.

### TNFα increases oxidative phosphorylation and biases astrocytes toward mitochondrial ATP production

**Figure 2:**
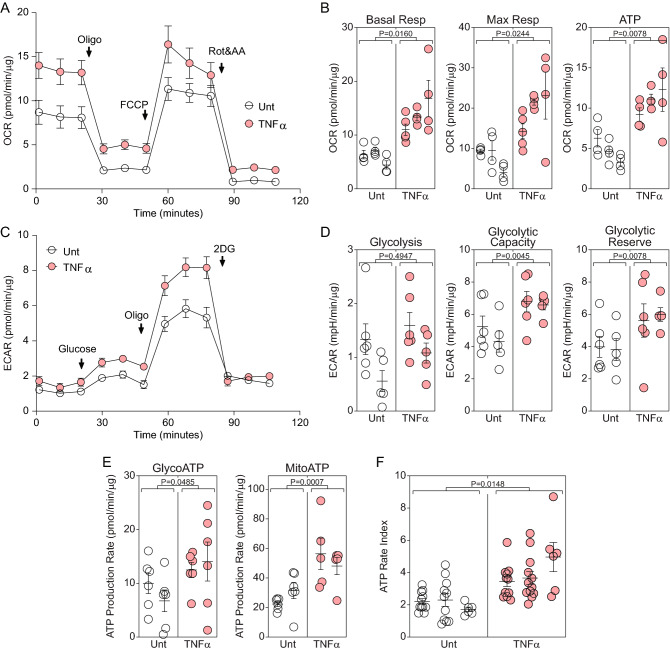
TNFα increases oxidative phosphorylation in quiescent astrocytes. (**A**) Astrocytes were left untreated (white symbols) or stimulated with TNFα (10 ng/mL for 24 h) (red) and then the realtime oxygen consumption rate (OCR) was measured using the Seahorse XF analyzer. Data are normalized to total protein (μg) present in the wells at the end of the analysis and represent at least 3 independent experiments. (**B**) Combined OCR measurements from technical replicates in 3 separate experiments are shown as mean ± SEM for the replicates; each symbol represents one well. P-values were calculated using mixed model nested t-tests. (**C**) Astrocytes were left untreated (white symbols) or stimulated with TNFα (10 ng/mL for 24 h) (red) and then the realtime extracellular acidification rate (ECAR) was measured. Data are normalized to total protein (μg) per well and represent at least 3 independent experiments. (**D**) Combined ECAR measurements from technical replicates in 2 separate experiments are shown as mean ± SEM for the replicates; each symbol represents one well. P-values were calculated using mixed model nested t-tests. (**E**) The contribution of glycolytic or mitochondrial ATP production was analyzed by Seahorse in quiescent cells stimulated with TNFα (10 ng/mL for 24 h) (red) or vehicle (DMSO; white). Data are normalized to total protein and shown as mean ± SEM from at least 6–8 replicates in two separate experiments. P-values were calculated using mixed model nested t-tests. (**F**) The ratio of mitochondrial ATP production rate to glycolytic ATP production rate was calculated in at least 8 technical replicates in 3 separate experiments and shown as mean ± SEM for the replicates. P-value was calculated using mixed model nested t-tests.

Previous work has shown that inflammatory cytokine stimulation converges on NFκB activation to drive downstream rewiring of the astrocytic bioenergetic phenotype^21^. To characterize the bioenergetic impact of TNFα on purified astrocytes under serum-free conditions we measured oxidative phosphorylation and ATP production using real-time extracellular flux analysis^22^. Cells stimulated with TNFα (10 ng/mL) for 24 h exhibited increased basal respiration, increased maximal respiration, and increased ATP production relative to vehicle (DMSO)-treated astrocytes using the oxygen consumption rate (OCR) normalized to total cellular protein (pmol/min/μg) (Fig. 2A, B). Measurement of the normalized extracellular acidification rate (ECAR) (mpH/min/μg) under the same conditions further revealed that TNFα stimulation increased the glycolytic reserve and glycolytic capacity of astrocytes but did not change the basal glycolytic rate (Fig. 2C, D). Analysis of the normalized ATP production rate (pmol/min/μg) mediated by glycolysis (glycoATP) versus oxidative phosphorylation (mitoATP) showed that TNFα increased both glycolytic and mitochondrial ATP production (Fig. 2E). However, taking the ratio of mitoATP to glycoATP, defined herein as the ATP rate index, shows that TNFα stimulation for 24 h increased overall astrocytic ATP production (Fig. 2F) by preferentially biasing the cell to produce ATP through enhanced oxidative phosphorylation.

### TNFα-induced Lcn2 secretion is ATP-dependent

**Figure 3:**
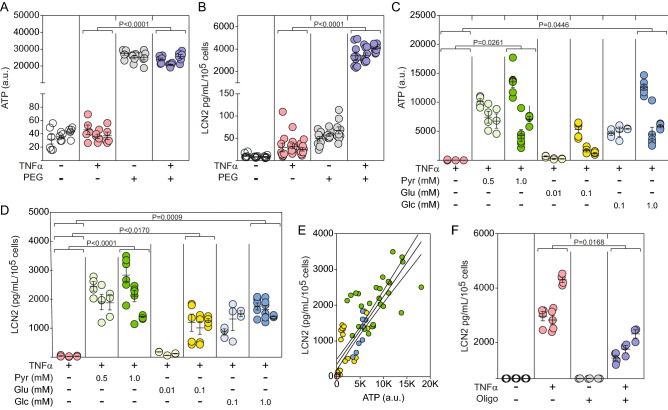
TNFα-induced Lcn2 secretion requires ATP primarily produced by oxidative phosphorylation. (**A**) Intracellular ATP levels were measured in astrocytes stimulated with TNFα (10 ng/mL for 24 h) in serum-free, phenol red-free media deprived of glucose (Glc), pyruvate (Pyr), and glutamate (Glu) or reconstituted with 1 mM pyruvate, 5 μM glutamate, and 1 mM glucose (PEG). Each symbol represents one well from 3 separate experiments, shown in nested layout. Mean ± SEM is shown for each biological replicate. P-values were calculated using mixed model nested t-tests. (**B**) Supernatants from the same cells in (**A**) were assessed for Lcn2 production by ELISA. Each symbol represents one well from 3 separate experiments, shown in nested layout. Mean ± SEM is shown for each biological replicate. P-values were calculated using mixed model nested t-tests. (**C**) Intracellular ATP levels were measured in astrocytes stimulated with TNFα (10 ng/mL for 24 h) in serum-free, phenol red-free media deprived of pyruvate (Pyr), glutamate (Glu), and glucose (Glc) individually reconstituted with each factor at different concentrations. Each symbol represents one well from 3 separate experiments, shown in nested layout. Mean ± SEM is shown for each biological replicate. P-values were calculated using mixed model nested ANOVA between treatment conditions. (**D**) Cell supernatants from the same cells in (**C**) were assessed for Lcn2 production by ELISA. Each symbol represents one well from 3 separate experiments, shown in nested layout. Mean ± SEM is shown for each biological replicate. P-values were calculated using mixed model nested ANOVA between treatment conditions. (**E**) Linear regression indicates that Lcn2 concentration is correlated with intracellular ATP concentration (R^2^ = 0.761) Solid line shows regression fit, with 95% CI shown as dotted lines. (**F**) Astrocytes were treated with the mitochondrial complex III inhibitor oligomycin (Oligo) (1 μM) for 30 min prior to stimulation with TNFα (10 ng/mL) for 24 h. Secreted Lcn2 was measured in supernatants by ELISA. Each symbol represents one well from 3 separate experiments, shown in nested layout. Mean ± SEM is shown for each biological replicate. P-values were calculated using mixed model nested ANOVA between treatment conditions.

Based on the TNFα-induced bias toward oxidative phosphorylation-derived ATP production, we hypothesized that astrocytic secretion of factors such as Lcn2 in response to TNFα is dependent upon ATP production. Given that glucose metabolism-derived metabolites enter the TCA cycle as pyruvate while glutamine is incorporated into the TCA cycle by conversion to α-ketoglutarate, we tested the TNFα-induced response in a TCA substrate-deficient environment. Astrocytes were incubated in DMEM lacking glucose (basal = 10 mM), pyruvate (basal = 1.3 mM), and glutamine (basal = 4 mM) media for 30 min and then resupplemented with pyruvate (Pyr or P: 0.5 or 1.0 mM), glutamate (Glu or E: 0.01 or 0.1 mM), and/or glucose (Glc or G: 0.1 or 1.0 mM) for 24 h in the presence or absence of TNFα (10 ng/mL) (PEG = Pyr + Glu + Glc). The total amount of cellular ATP in the absence of TCA substrates was decreased from 26,087 units to 37 units and stimulation with TNFα did not alter this difference (Fig. 3A). Notably, the absence of TCA substrates reduced basal secretion of Lcn2 from 58 to 10 pg/mL/10^5^ cells and profoundly suppressed TNFα-induced Lcn2 release from 3642 pg/mL/10^5^ cells in the resupplemented media to 30 pg/mL/10^5^ cells in the deficient media (Fig. 3B). Adding back individual substrates revealed that each factor was sufficient to at least partially restore ATP levels, although glutamate was less effective than either pyruvate or glucose at the concentrations utilized (Fig. 3C). In parallel, each factor was sufficient to restore Lcn2 secretion in response to TNFα stimulation, with pyruvate providing the most robust response (Fig. 3D). The relationship between substrate-induced ATP restoration and TNFα-induced Lcn2 release indicates that the inflammatory response scales with the amount of cellular ATP (R^2^ = 0.76) (Fig. 3E). Finally, pharmacological inhibition of oxidative phosphorylation-derived ATP synthesis with oligomycin (1 μM) reduced Lcn2 production in response to TNFα from 3000 pg/mL/10^5^ cells to 1281 pg/mL/10^5^ cells (Fig. 3F). These findings indicate that TNFα-induced Lcn2 secretion by astrocytes requires ATP and that oxidative phosphorylation is a primary source of this energy substrate.

### Teriflunomide biases astrocytic metabolism toward glycolytic ATP production

**Figure 4:**
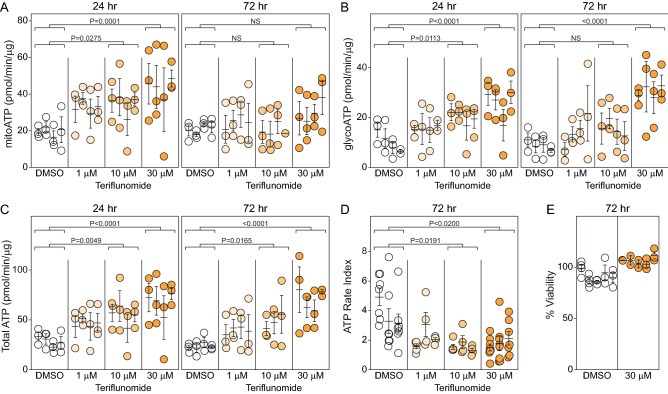
Teriflunomide increases glycolysis in quiescent astrocytes. Astrocytes grown under serum-free conditions were treated with teriflunomide (1, 10, 30 μM) under two paradigms. In the first, cells were incubated for 24 h. In the second, cells were treated once, then again after 48 h, and analyzed 24 h later (72 h total). Mitochondrial ATP production rate (**A**), glycolytic ATP production rate (**B**), and total ATP production rate (**C**) were measured using the Seahorse XF analyzer. Data are normalized to total protein (μg) per well. Each symbol represents one well from 3 separate experiments, shown in nested layout. Mean ± SEM is shown for each biological replicate. P-values were calculated using mixed model nested ANOVA between treatment conditions. (**D**) The ratio of mitochondrial ATP production rate to glycolytic ATP production rate was calculated from the data in (**A**) and (**B**) to indicate the ATP rate index in the 72 h paradigm. Each symbol represents one well from 3 separate experiments, shown in nested layout. Mean ± SEM is shown for each biological replicate. P-values were calculated using mixed model nested ANOVA between treatment conditions. (**E**) Viability of astrocytes exposed to teriflunomide (30 μM) in the 72 h paradigm was measured by MTT assay. Data are normalized to vehicle-only. Each symbol represents one well from 3 separate experiments, shown in nested layout. Mean ± SEM is shown for each biological replicate.

Based on recent evidence that the DHODH inhibitor teriflunomide (TF) preferentially modulates autoreactive T cells by suppressing oxidative phosphorylation^18^, we hypothesized that this drug would reduce the astrocytic inflammatory response to TNFα stimulation. To establish the influence of TF on pure, quiescent astrocytes, the cells were stimulated with TF (1, 10, 30 μM) for 24 h (TF added at 0 h) or 72 h (TF added at 0 and 48 h) in the absence of TNFα and then ATP production was assessed via oxidative phosphorylation or glycolysis. While a single exposure to 30 μM TF increased the mitochondrial ATP production rate at 24 h, this effect was not sustained at 72 h following a second exposure for 24 h (Fig. 4A). In contrast, glycolytic ATP production was increased at 24 and 72 h in response to 30 μM TF (Fig. 4B). This effect was confirmed by ECAR measurement, which showed that TF increased basal glycolysis and glycolytic capacity in the astrocytes (Supplemental Fig. 1). Moreover, total cellular ATP production rate was increased at both 24 and 72 h in response to either 10 or 30 μM TF (Fig. 4C). Calculating the ATP rate index at 72 h, as above, revealed that even 1 μM TF trended toward shifting astrocytic metabolism toward glycolytic ATP production relative to DMSO (Fig. 4D; DMSO vs 1 μM TF, P = 0.0899). This effect was not associated with increased cellular toxicity, as all 3 TF concentrations preserved viability at or above vehicle control levels (Fig. 4E; 30 μM shown). We conclude that teriflunomide increases both mitochondrial and glycolytic ATP production, but that the predominant effect at 72 h is a skew toward glycolytic production. Indeed, 10 μM TF reduced the ATP rate index to less than 1.3 (relative to almost 4 for the vehicle control), indicating near equalization of the contribution of glycolytic ATP production and oxidative phosphorylation production (Fig. 4D).

### Teriflunomide preconditioning blocks the bioenergetic impact of TNFα and reduces the astrocytic inflammatory response

**Figure 5:**
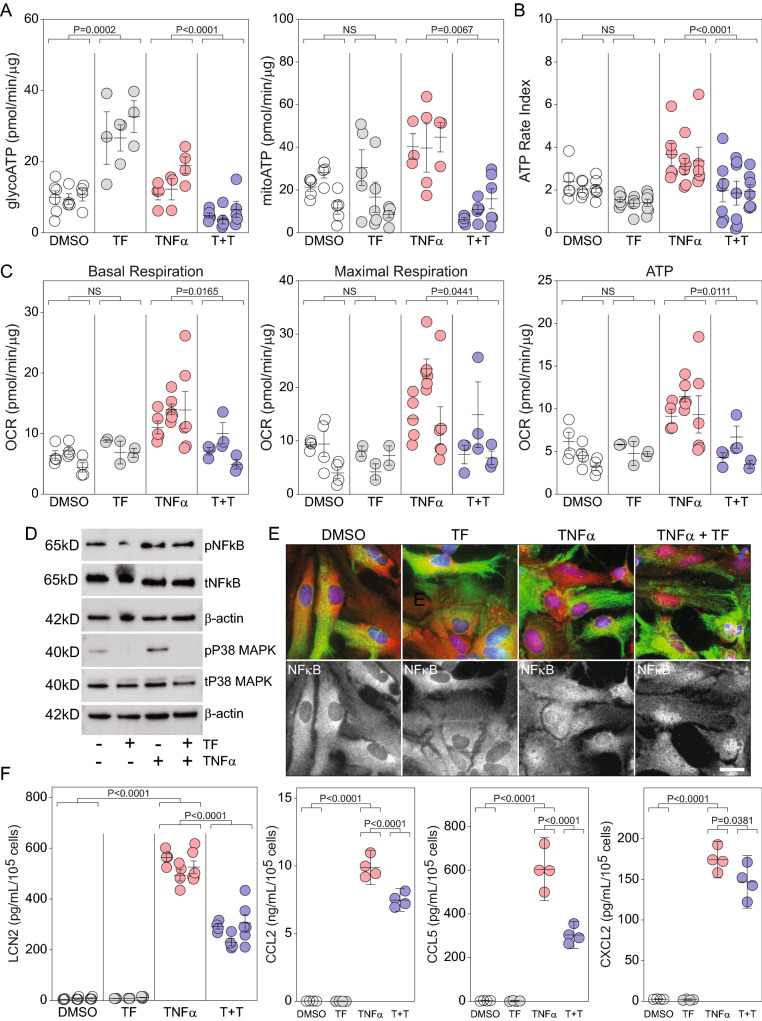
Teriflunomide preconditioning blocks the bioenergetic effect of TNFα and inhibits release of reactive inflammatory factors. Quiescent astrocytes were preconditioned with teriflunomide (TF; 30 μM) using the 72 h treatment paradigm. TNFα (10 ng/mL) was added at the same time as the final teriflunomide treatment and glycolytic and mitochondrial ATP production (**A**), the ATP rate index (**B**), and the oxygen consumption rate (**C**) were measured 24 h later. T + T = TF + TNFα condition. Each symbol represents one well from 3 separate experiments, shown in nested layout. Mean ± SEM is shown for each biological replicate. P-values were calculated using mixed model nested ANOVA between treatment conditions. TF reduced the TNFα-induced increase in all assays to control (DMSO) levels (glycoATP: T + T vs DMSO, P = 0.1338; mitoATP: T + T vs DMSO, P = 0.5020; basal respiration: T + T vs DMSO, P = 0.8059; maximal respiration: T + T vs DMSO, P = 0.9588; ATP: T + T vs DMSO, P = 0.9935). Preconditioning with teriflunomide did not inhibit NFκB phosphorylation (**D**) or nuclear translocation (**E**) in response to TNFα stimulation. Immunoblots for NFκB derive from the same transfer membrane. Immunoblots for p38 derive from the same membrane. Uncropped blots are available in the Supplementary Information. p38 phosphorylation was completely abrogated by teriflunomide preconditioning. Upper panels in (**E**) show GFAP (green), NFκB (red), and DAPI (blue); lower panels show specific NFκB signal. (**F**) Teriflunomide preconditioning reduced Lcn2, CCL5, CCL2, and CXCL1 release from astrocytes stimulated with TNFα (10 ng/mL). Each symbol represents one well from 3 separate experiments, shown in nested layout. Mean ± SEM is shown for each biological replicate. P-values were calculated using mixed model nested ANOVA between treatment conditions. Scale bars are 20 μm.

The bias toward glycolytic ATP production induced in astrocytes at 72 h by TF suggested that this inhibitor might counter the oxidative phosphorylation skew driven by TNFα. Using a preconditioning paradigm in which astrocytes were treated with TF (30 μM) at -48 h and then treated again with TF (30 μM) and TNFα (10 ng/mL) at 0 h, revealed that 24 h later the cellular bioenergetic profile was renormalized relative to TNFα alone. While TF alone persistently increased glycoATP and TNFα alone biased toward mitoATP, astrocytes that were preconditioned with TF and then stimulated with TNFα exhibited glycoATP and mitoATP production rates that were not different from vehicle control (Fig. 5A) (glycoATP: T + T vs DMSO, P = 0.1338; mitoATP: T + T vs DMSO, P = 0.5020). This renormalization effect is further evident in the ATP rate index, which shows that TF pushed TNFα-induced ATP production back to control levels (Fig. 5B) (ATP rate index: T + T vs DMSO, P = 0.8638). TF preconditioning also blocked the TNFα-induced increase in oxidative phosphorylation (Fig. 5C), with normalization of basal respiration, maximal respiration, and ATP production to vehicle control levels (basal respiration: T + T vs DMSO, P = 0.8059; maximal respiration: T + T vs DMSO, P = 0.9588; ATP: T + T vs DMSO, P = 0.9935). The TF effect was not mediated by inhibition of NFκB, as phosphorylation and nuclear translocation of this signaling molecule were unchanged relative to TNFα alone (Fig. 5D, E). Notably, phosphorylation of p38 MAPK in response to TNFα was completely prevented by TF preconditioning (Fig. 5D). Finally, TF preconditioning significantly reduced the inflammatory response to TNFα, with a 40% reduction in Lcn2 secretion, a 40% reduction in CCL5, a 25% reduction in CCL2, and a 15% reduction in CXCL2 (Fig. 5F). These results indicate that teriflunomide renormalizes the astrocytic bioenergetic response to TNFα, resulting in a reduced inflammatory response.

## Discussion

Immunomodulation via metabolic reprogramming and control of bioenergetic pathways has revolutionized our approach to therapy in numerous immune-mediated diseases^23^. Notably, however, many of these therapies impose control by inhibiting resources required for the robust proliferative response induced by inflammatory and autoimmune drivers^23,24^. For example, teriflunomide-induced inhibition of DHODH limits de novo pyrimidine biosynthesis and thereby reduces T cell proliferation^25^, resulting in improved outcomes in patients with relapsing–remitting multiple sclerosis^18^. Teriflunomide also depletes ATP in highly proliferative cells^26^ and interferes with complex III of the mitochondrial electron transport chain, reducing T cell activation in response to proliferative drivers^18^. Indeed, most immune cells, per se, share a common proliferative response to inflammatory activators that results in a shift from basal oxidative metabolism to aerobic glycolysis^27–29^.

In contrast to immune cells such as lymphocytes, which have relatively minimal basal metabolic demands prior to induction of proliferation and activation^30^, astrocytes under homeostatic conditions bear a large and critical metabolic burden^31^. Astrocytes support the energy demands of neurons and neural circuits by providing lactate generated through the metabolism of glucose to pyruvate in the glycolytic pathway. Astrocytes also support neurotransmitter homeostasis and network function via the ATP-dependent conversion of extracellular glutamate into glutamine that is released for neuronal uptake and reprocessing^32^. At the same time, astrocytes respond to inflammatory drivers using mechanisms that are shared with immune cells^33^. However, it is only under the most profound injury conditions that astrocytes proliferate in response to these drivers^34^. This dichotomy suggests that metabolic modulation of astrocytes may involve processes and responses that are fundamentally different from immune cells such as lymphocytes.

Exploring the specific immunometabolism of astrocytes requires careful consideration of the system used for analysis. To date, the majority of work on astrocyte metabolism has involved two problematic components that limit interpretability and mechanistic understanding. The first issue is the use of FBS as a source of trophic support in glial cultures. Given that FBS itself drives inflammatory activation of astrocytes^35^, the analysis of metabolic pathways induced by inflammatory drivers in the presence of FBS is compromised by the absence of a metabolically quiescent baseline. The second problem is the assignment of inflammatory and metabolic responses to astrocytes in culture models that are better characterized as mixed glia^36^. Most studies employ shake-off cultures that enrich astrocytes by physically displacing microglia and oligodendrocytes from a firmly adherent astrocyte monolayer. However, this method only results in a reduction in microglia, not complete ablation. Therefore, in this study we have effectively completely ablated microglia by treating post-shake-off cultures with clodrosome^19^ and we have cultured the highly purified astrocytes (> 98.8% ASCA-2^+^ astrocytes, < 0.1% CD11b^+^ microglia, < 0.3% O4^+^ oligodendrocytes) in long-term serum-free conditions that promote a quiescent baseline.

Using this system, we demonstrated that stimulation with TNFα drives astrocytic production of Lcn2 and other inflammatory factors and simultaneously increases both oxidative phosphorylation and glycolysis, with a robust skew toward mitochondrial ATP production. We further showed that the Lcn2 response induced by TNFα is dependent on ATP. However, blocking the electron transport chain with oligomycin only attenuated Lcn2 release by approximately 50%, indicating that astrocytes maintain a glycolytic component of ATP production even under inflammatory drive. The observation that ATP production and the Lcn2 response to TNFα is reconstituted, at least in part, by reintroduction of either pyruvate, glutamate, or glucose indicates that astrocytes have a high degree of metabolic substrate flexibility. Despite the homeostatic energy differences between astrocytes and lymphocytes and the differential proliferative response induced by inflammation, these findings suggest that both cell types engage oxidative phosphorylation-dependent ATP production as part of the response to inflammatory drivers.

Under quiescent conditions, we found that astrocytes generate approximately 20 pmol/min/μg ATP from oxidative phosphorylation and approximately 10 pmol/min/μg via glycolysis. Incubation of these homeostatic cells with teriflunomide for 24 h increased ATP production via both pathways in a dose-dependent manner, doubling the mitochondrial ATP output to about 40 pmol/min/μg and more than doubling the glycolytic output to about 25 pmol/min/μg. In itself, this suggests that teriflunomide induces an overall enhancement of astrocytic bioenergetic output, a finding that may have relevance to therapeutic interventions in neurodegenerative conditions associated with compromised ATP production^37,38^. However, the impact of teriflunomide on oxidative phosphorylation was not maintained when cells were stimulated with the drug and then restimulated 48 h later and assessed at 72 h after the initial exposure. Instead, this paradigm resulted in continued enhancement of glycolytic ATP production, increasing the rate to 30 pmol/min/μg. Calculating the ratio between mitochondrial and glycolytic ATP production rate revealed that teriflunomide skewed the basal metabolism of astrocytes from a state in which oxidative phosphorylation output dominated glycolytic output by over 2:1 to a state in which glycolytic output equaled mitochondrial production. Moreover, this effect occurred even at low doses and was associated with increased glycolytic capacity and glycolytic reserve but unchanged basal or maximal respiration. This outcome is sharply different from the naive T cell response to teriflunomide, in which the drug does not alter mitochondrial respiration or glycolytic capacity over 72 h^18^.

Teriflunomide also robustly altered the astrocytic bioenergetic response to TNFα stimulation, though the nature of this effect was different from the lymphocyte response to the drug. Teriflunomide essentially renormalized ATP production in astrocytes, returning both glycolytic and mitochondrial rates to below baseline levels and restoring the ratio of mitochondrial to glycolytic production to more than 2:1. The drug also returned basal and maximal respiration to baseline levels. In contrast, teriflunomide reduced these measures in stimulated T cells but did not return the cells to baseline levels^18^. Unexpectedly, despite the nearly complete renormalization of metabolism, teriflunomide only partially inhibited Lcn2 and chemokine production induced by TNFα stimulation. Notably, despite evidence that TF inhibits NFκB signaling in some contexts^16^, we observed no reduction in TNFα-induced NFκB phosphorylation or nuclear translocation in astrocytes preconditioned with TF. However, TF completely inhibited p38 phosphorylation in response to TNFα stimulation and reduced phospho-p38 levels below baseline in unstimulated astrocytes. Given the TF-induced skew in basal metabolism toward enhanced glycolytic ATP production under non-inflammatory conditions and the induction of mitochondrial ATP production in response to TNFα, these findings suggest that p38 activation may depend upon oxidative phosphorylation.

The p38 serine-threonine kinase is a mitogen-activated protein kinase (MAPK) that is proximally activated by the dual-specificity MAPK kinases MKK3 and MKK6^39^. p38 is a critical regulator of cellular stress responses, driving specific signaling cascades involved in activation, differentiation, apoptosis, and autophagy in cells stressed by cytokines, radiation, osmotic shock, heat shock, and oxidative stress^40^. These cascades converge on MKK3/6 and p38 downstream from a diverse range of MAPK kinase kinases. One such upstream kinase is the apoptosis signal-related kinase ASK1, which is activated by both oxidative stress^41^ and TNFα^42^ via increased intracellular reactive oxygen species (ROS) production^43^. TNFα-induced mitochondrial ATP production results in generation of ROS^44^, oxidation of thioredoxin^45^, and consequent derepression of ASK1 activity^46^, leading to p38 activation^42^. In turn, the primary downstream target of p38, the MAPK-activated protein kinase 2 (MAPKAPK2), is involved in post-transcriptional regulation of numerous inflammatory and stress response mRNAs. The likely mechanism for post-transcriptional control by MAPKAPK2 is stabilization of mRNAs bearing adenosine/uridine-rich repeat elements (AREs) such as AUUUA^47^—in the absence of MAPKAPK2 signaling these AREs destabilize transcripts and shorten half-life, reducing the pool of mRNA available for translation^48^. Notably, Lcn2 has a canonical AUUUA motif within an intron that is conserved across splice variants^49,50^. In this context it is therefore interesting that TF-treated astrocytes failed to show a sustained increase in Lcn2 RNA after stimulation for 24 h with TNFα (TNFα only = sixfold induction; TNFα + TF = 0.9-fold induction). Given the incomplete inhibition of Lcn2 release in TF-treated astrocytes, this suggests that an initial wave of inflammatory factor release occurred in response to TNFα, but continued production and release was thwarted by degradation of the RNA encoding these factors. Furthermore, the inability to maintain the inflammatory response due to RNA degradation is consistent with the observation that TF does not prevent NFκB activation. Thus, in TF-treated astrocytes stimulation with TNFα may drive signaling through NFκB, engaging the machinery necessary to generate inflammatory mRNAs, but the absence of p38 signaling results in failure to stabilize these RNAs, thereby limiting translation and production of additional protein. Further research is necessary to test this hypothesis, but one intriguing speculation that arises from this concept is that TF may serve to more broadly impede inflammatory responses associated with p38 signaling, such as the senescence-associated secretory pathway or neuroinflammation associated with seizures and psychiatric disorders.

In conclusion, using highly purified astrocytes grown under conditions that preserve a non-reactive state, we found that TNFα drives inflammatory signaling and biases the cellular bioenergetic profile toward mitochondrial ATP production. In contrast, teriflunomide biases astrocytic metabolism toward glycolytic ATP production. Preconditioning with teriflunomide prevents the TNFα-induced bioenergetic shift, resulting in maintenance of homeostatic ATP production. Consequently, the astrocytic inflammatory response is reduced. These findings suggest that teriflunomide impacts metabolism in astrocytes in a manner that may reduce neuroinflammation across a spectrum of neurological diseases.

## Materials and methods

### Mice

No experiments were performed on live animals. C57Bl/6 mice were used for preparation of primary astrocytes, as described below. Neonatal animals were not sexed prior to euthanasia. Neonatal mice were killed by decapitation, as per the AVMA Guidelines for the Euthanasia of Animals and the ACLAM Task Force on Rodent Euthanasia. All studies were conducted in accordance with the United States Public Health Service’s Policy on Humane Care and Use of Laboratory Animals and were approved by the Mayo Clinic Institutional Animal Care and Use Committee (Animal Welfare Assurance number A3291-01). Where relevant, experiments and procedures conformed to ARRIVE guidelines.

### Mouse cortical primary astrocyte cultures

Primary murine astrocyte cultures were prepared from P1-P3 C57BL/6J mouse pups, as described^51^. Briefly, pups were individually separated from the dam, wiped quickly with 70% EtOH, and decapitated. The skull was opened with forceps and the brain was removed into PBS. The olfactory bulbs and cerebellum were removed, the cortices were separated, the inner face of each cortex was scooped clean with forceps, and meninges were removed from the cortical surface. After collecting all cortices, the pooled material was washed in PBS, minced into 1 mm^2^ blocks with a sterile single-edge blade, and digested with 0.25% trypsin (Invitrogen, 15090-046, no phenol red) prepared in Earle's balanced salt solution (EBSS; NaCl 7.02 g/L, NaH_2_PO_4_-H_2_O 0.84 g/L, KCl 0.14 g/L, glucose 3.60 g/L, NaHCO_3_ 0.84 g/L) containing HEPES (4.80 g/L) and bovine serum albumin (BSA; 3.00 g/L) for 30 min at 37 °C with shaking at 80 rpm. Trypsin was quenched with 10% heat-inactivated fetal bovine serum (FBS) and the suspension was incubated on ice for 5 min with 0.08% MgSO4 and DNase I (0.02 mg/mL). Tissue pieces were pelleted at 200 g for 5 min, triturated into a single cell suspension, resuspended in EBSS containing HEPES and BSA, underlaid with 1.25 mL 4% BSA prepared in EBSS, and centrifuged at 100 g for 8 min without braking. The cell pellet was resuspended in astrocyte growth media (AGM; high glucose Dulbecco’s modified eagle medium (DMEM, VWR, 45000-304); 10% FBS (Sigma, 12306C); 1% penicillin–streptomycin (Gibco, 15,140–122)) and cells were plated at 1.3 × 10^5^ cells/cm^2^ on T75 flasks coated with poly-l-lysine hydrobromide (Sigma, P1524). After 4 days, flasks were shaken at 200 rpm at 37˚C with 5% CO_2_ to remove microglia and oligodendroglia. Growth media was replaced after 24 h and shake-off continued for another 24 h. Pure astrocytes were obtained by treating the subsequent enriched cultures with 100 μg/mL clodrosome (Encapsula Nanoscience, 8909) for 72 h^19^. On day 12, cells were washed with AGM containing 5 ng/mL heparin binding epidermal growth factor (HbEGF; PeproTech, 100-47) with no FBS. This serum-free media was continuously refreshed every 48–72 h. On day 25 in vitro, cells were lifted with 0.25% trypsin–EDTA (Gibco, 25200-056) and replated at 5.2 × 10^4^ cells/cm^2^ into wells coated with poly-d-lysine hydrobromide (Sigma, P1024). Trypsin was neutralized with 10% FBS but was washed off immediately with serum-free AGM-HbEGF before plating. Cells were used for experiments after 28 days in vitro, or 3 days post-plating.

### Astrocyte purity assay

Cells were scraped into cold PBS, pelleted, and resuspended in 4% paraformaldehyde for 10 min. After washing in PBS, cells were resuspended in blocking buffer (PBS containing 1% BSA and ~ 0.1 μg/μL 2.4G2 anti-mouse CD16/CD32 antibody) for 60 min on ice. Cells were pelleted and resuspended in primary antibody solution (PBS plus 1% BSA) for 60 min at RT. Following washing labeled cells were analyzed by flow cytometry using an Attune NxT instrument (Invitrogen). Primary antibodies were: APC-conjugated anti-astrocyte cell surface antigen-2 (ASCA-2) antibody (clone IH3-18A3; Miltenyi Biotec #130-099-138); PE-conjugated O4 antibody (Miltenyi Biotec #130-115-810); FITC-conjugated anti-CD11b antibody (BD Biosciences #557396). All were used at 1:100 dilution. Cells were counted and individual population numbers were determined as percent of total.

### (3-(4,5-Dimethlythiazolyl-2)-2,5-diphenyltetrazolium bromide (MTT) viability assay

Cells (8 × 10^4^) were plated in a 48-well dish and allowed to reach confluency. Light-sensitive yellow tetrazolium MTT (3-(4,5-dimethlythiazolyl-2)-2,5-diphenyltetrazolium bromide, Sigma, M2128) was then added at a 1:10 dilution cells were incubated at 37 °C, 5% CO_2_ for 1 h. Metabolically active cells reduced the MTT through interaction with NADH and NADPH, resulting in formation of an intracellular purple formazan precipitate. After aspiration of the media using a fine tip, 400 µL of SDS-DMF was added to lyse the cells and solubilize the formazan. After pipetting up a down, 100 µL of each sample was transferred to each of 2 duplicate wells of a 96-well plate and placed on a shaker for 15 min to promote dissolution. Formazan was quantified by spectrophotometric readings at 570 nm on a SpectraMax M3 spectrophotometer (Molecular Devices) using SoftMax Pro Software.

### Enzyme linked immunosorbent assay (ELISA) detection

Following stimulation, supernatants were collected, clarified by centrifugation, and held at -80 °C until analysis via ELISA. Lcn2 and CCL5 were measured in supernatants using a mouse Lcn2 ELISA assay kit (R&D, DY1857) and a mouse CCL5/RANTES ELISA assay kit (Antigenix, RMF432CK). Mouse CCL2 (RMF426CKC) and CXCL1 (RMF428CKC) were also measured in the supernatants using ELISA construction kits (Antigenix America).

### Adenosine triphosphate (ATP) detection assay

ATP was measured using the CellTiter-Glo^®^ 2.0 assay kit (Promega G9241) while keeping the cell number constant. Briefly, astrocytes were seeded at 35,000/well into a black clear bottom 96 well plate. After treatment, an equivalent volume of CellTiter-Glo® 2.0 reagent was added to the well without washing off media. Reactions were incubated at room temperature for 2 min on a shaker. The plate bottom was sealed with white tape. Luminescence was measured using a SpectraMax M3 spectrophotometer (Molecular Devices) with 500 ms integration.

### Multiplexed cytometric bead array (CBA)

The mouse MCP-1 flex set (BD 558342), mouse KC flex set (BD 558340), and mouse RANTES flex set (BD 558345) were used according to the manufacturer's instructions. 50 µl of cell supernatant was assayed against a standard curve using an Accuri C6 (BD Biosciences) flow cytometer.

### Extracellular flux analysis

Astrocytes (10^5^ cells/well for 24 well plate or 35,000/well for 96 well plate) were seeded on to XF Seahorse plates (Agilent #102340-100, #102416-100). For both glycolysis (glyco) and mitochondrial (mito) stress analyses, media conditions were extensively optimized for astrocytes. Seahorse cartridges were pre-equilibrated one day before extracellular flux analysis using XF calibrant (pH 7.4; Agilent #100840-000) at 37 °C in a non-CO_2_ incubator. On the day of the experiment, we added XF-DMEM media (Agilent #103575-100) containing 180 µM sodium pyruvate (Sigma #S8636), 0.5 mM glutamine (Gln; Gibco #2503008), 5 µM glutamate (Glu; Sigma #PHR2634), and 10 mM glucose (Sigma #G8769; for mito stress only, as the glycolysis stress test delivers the same concentration of glucose during the assay) to the cells just before incubation at 37 °C for 1 h. For glycolysis stress analysis, glucose (10 mM), oligomycin (3 µM; Sigma #75351) and 2-DG (50 mM; Cayman #14325) were injected via port A, B and C respectively. For the mitochondrial stress assay oligomycin (3 µM), FCCP (1.5 µM; Sigma #C2920) and rotenone (Sigma #R8875) + antimycin A (Sigma #A8674) (1 µM + 1 µM) were injected via port A, B and C respectively. Real-time ATP production rate was assessed by analyzing extracellular flux in response to 3 µM oligomycin and 1 µM rotenone + antimycin A (1:1) injected via port A and B respectively. Total protein per well was quantified by BCA analysis after the seahorse experiment and these values were used to normalize the bioenergetic measurements. All analyses were performed in Wave software and then exported to Excel and GraphPad Prism 8.0 for further analysis.

### Western blot assay

Astrocytes were grown to 90–100% confluency and total protein was harvested using RIPA (Sigma, R0278) or NP40 lysis buffer (Life technologies, FNN0021) containing 1 mM PMSF (Fisher NC9953637), protease inhibitor tablet (Thermo Fisher, 88668), and 10 mM sodium butyrate (Cayman, 13121). Insoluble cell debris was removed by centrifugation. Lysates were stored at − 20 °C. Protein quantification was performed using Pierce BCA Protein Assay Kit (Thermo Fisher, 23227). All samples were diluted 1:10 and absorbance was read at 570 nm. Samples were resolved on 5–20% Mini-PROTEAN precast protein gels (BioRad, 4561095) at 134 V for 1 h. Proteins were transferred onto PVDF membrane (104 V for 1 h at 4 °C). Membranes were blocked with 5% milk (5 g in 100 mL tris-buffered saline containing 0.1% Tween-20 (TBST)) for 1 h, washed with TBST, and incubated with primary antibody at 1:1000 overnight at 4 °C. Concentrations of the primary antibodies utilized were: phospho-NFκB (1:1000, Cell Signaling, 3033), total NFκB (1:1000, Cell Signaling, 8242), β-actin (1:1000, Cell Signaling, 4967). Membranes were washed three times with TBST (15 min incubation at room temperature on rocker), and horseradish peroxidase (HRP)-labeled secondary antibodies were applied at 1:5000 dilution at room temperature for 1 h on the rocker. Blots were developed with West Pico TMB/H_2_0_2_ substrate and protein bands were visualized using a Chemidoc and/or Kodak imager with film. Original blots are shown in Supplementary Information.

### Imaging

Bright field images were taken using an Olympus CKX41 microscope. Images were captured with CellSens standard software. For immunofluorescent images astrocytes were seeded in 4- or 8-well chamber slides (Millipore Sigma, C6932) coated with PDL and 20 μg/mL laminin I (Culturex, 3400-010-02). Cells were fixed in 100% methanol at − 20 °C for 5 min. After brief PBS wash, cells were blocked in 3% BSA for 1 h followed by overnight primary antibody incubation at 4 °C. Primary antibodies were: mouse anti-GFAP (1:1000; EMD Millipore MAB360); rabbit anti-NFκB (1:1000, Cell Signaling #8242). After extensive washing, secondary antibodies were added for 1 h at room temperature (1:1000 dilution). Nuclei were counterstained with DAPI containing mounting media (ThermoFisher Scientific, P36962). Images were acquired on a Zeiss Axio Observer Z1. Image J software was used for quantification.

### Experimental design and statistical analysis

Experiments were performed in at least 3 separate biological replicates (cultures) prepared from independent animal cohorts. Where applicable, a minimum of 3 technical replicates were utilized in each experiment. A nested biological:technical replicate design was employed for all relevant graphs and statistical analyses. Mixed model t-tests were used to calculate significance for experiments with one treatment; mixed model one-way ANOVA was used to for nested designs with multiple treatments. Data from each biological replicate are presented as mean ± SEM of the technical replicates. P-values less than 0.05 were considered significant. Both Microsoft Excel and GraphPad Prism 8 (GraphPad Software, Inc.) were used to analyze the data.

### Ethics approval

All studies were conducted in accordance with the United States Public Health Service’s Policy on Humane Care and Use of Laboratory Animals and with approval from the Mayo Clinic Institutional Animal Care and Use Committee.

## Supplementary Information

Supplementary Information.

Supplementary Information.

## Data Availability

The datasets used and/or analyzed during the current study are available from the corresponding author on reasonable request.

## Abbreviations

Lcn2: Lipocalin-2
TF: Teriflunomide
DHODH: Dihydroorotate dehydrogenase
GFAP: Glial fibrillary acidic protein
TNFα: Tumor necrosis factor alpha
OCR: Oxygen consumption rate
ECAR: Extracellular acidification rate
TCA: Tricarboxylic acid
FBS: Fetal bovine serum
ASCA-2: Astrocyte cell surface antigen-2
MAPK: Mitogen-activated protein kinase
ASK1: Apoptosis signal-related kinase
ROS: Reactive oxygen species
