# Supplementary material for: Teriflunomide shifts the astrocytic bioenergetic profile from oxidative metabolism to glycolysis and attenuates TNFα-induced inflammatory responses
Source: Sci Rep. 2022 Feb 23;12:3049. doi: 10.1038/s41598-022-07024-7 (PMC8866412; doi:10.1038/s41598-022-07024-7)
Supplement: Supplementary file 1 — Supplementary Information. [file 41598_2022_7024_MOESM1_ESM.pdf]

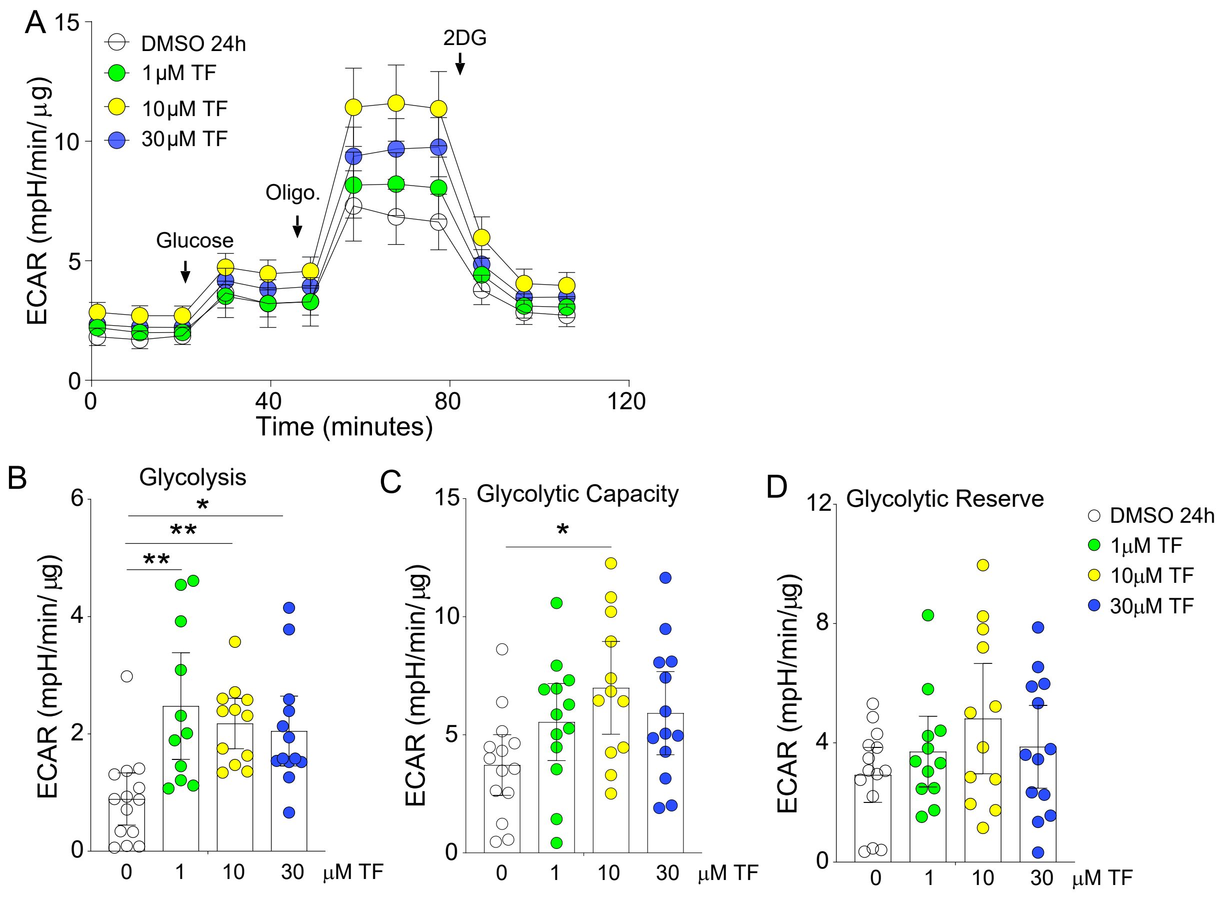

(A) Real-time extracellular acidification rate (ECAR) measurement of astrocytes after DMSO, 1 μM, 10 μM and 30 μM TF treatment for 24h. Data were normalized against total protein (μg). XF-base media re-constituted with 180 μM pyruvate, 5 μM glutamate, 500 μM glutamine and pH adjusted to 7.4 was used for this analysis. Quantification of basal glycolysis (B), glycolytic capacity (C), and glycolytic reserve (D) in response to different TF concentrations for 24h. Each bar is reported as the mean ± SD. Two-way ANOVA was performed to detect the significant comparison between treatments followed by Holm-Sidak's correction. *, $p < 0.05$; **, $p < 0.01$ versus untreated condition.
