# Supplementary figures and images for: Teriflunomide shifts the astrocytic bioenergetic profile from oxidative metabolism to glycolysis and attenuates TNFα-induced inflammatory responses
Source: Sci Rep. 2022 Feb 23;12:3049. doi: 10.1038/s41598-022-07024-7 (PMC8866412; doi:10.1038/s41598-022-07024-7)

1C

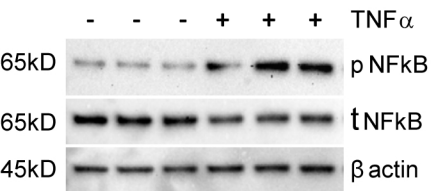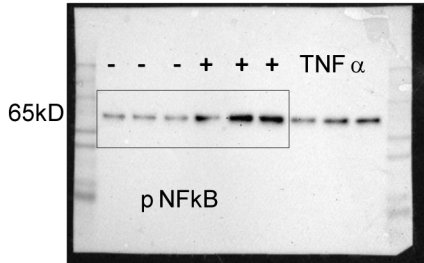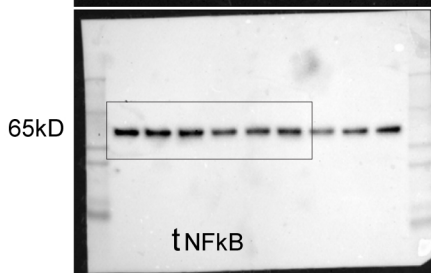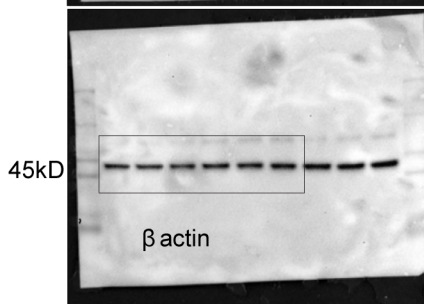

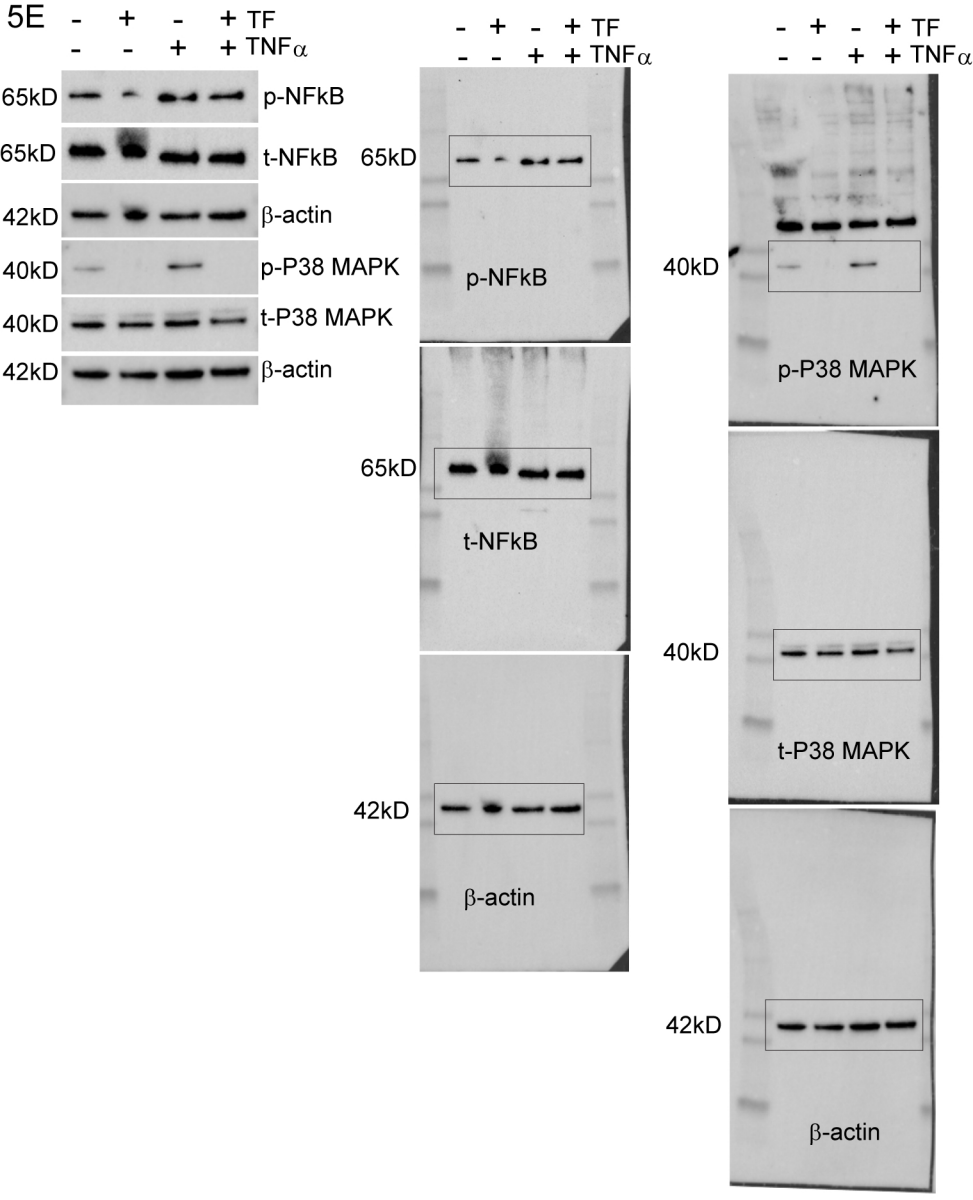

Supplement: Supplementary file 2 — Supplementary Information. [file 41598_2022_7024_MOESM2_ESM.pdf]
